# Knowledge and associated factors of patients toward informed consent in obstetric and gynecologic surgical procedures at Debre Markos Comprehensive Specialized Hospital, Ethiopia

**DOI:** 10.3389/fsurg.2025.1472033

**Published:** 2025-06-05

**Authors:** Addisu Andualem Ferede, Mamaru Getie Fetene, Endinew Beka Mehiretie, Worku Taye Getahun, Aysheshim Asnake Abneh

**Affiliations:** ^1^ Department of Midwifery, College of Medicine and Health Science, Debre Markos University, Debre Markos, Ethiopia; ^2^ Department of Midwifery, Debre Markos Comprehensive Specialized Hospital, Debre Markos, Ethiopia; ^3^ Department of Public Health, College of Medicine and Health Science, Debre Markos University, Debre Markos, Ethiopia

**Keywords:** knowledge, obstetric and gynecologic, surgical informed consent, women, Ethiopia

## Abstract

**Background:**

Informed consent (IC) is a principle in medical ethics and medical law that a patient must have adequate information and understanding before making decisions about their medical care. It is very essential for both clinician and patient in surgery and should be seen as a usual activity. Knowledge is one of the main intervention tools to improve patient comprehension in informed consent for surgical patients. In Ethiopia, little is known about the knowledge level and its determinant factors toward obstetric and gynecologic surgical informed consent. Due to this reason, this study aimed to assess the level of knowledge and associated factors of surgical informed consent among patients who underwent obstetric and gynecologic surgery at Debre Markos Comprehensive Specialized Hospital.

**Methods:**

An institutional-based cross-sectional study was conducted from 28 November to 30 December 2023, among 298 postoperative women admitted to Debre Markos Comprehensive Specialized Hospital. Data were collected using a pretested, structured interviewer-administered questionnaire by using a systematic random sampling technique. The collected data were entered into EpiData version 4.6 and then exported to SPSS statistical software version 25 for analysis. A multivariable logistic regression analysis was employed to estimate the effect of independent variables on the outcome variable. An adjusted odds ratio [adjusted odds ratio (AOR)] with a 95% confidence interval (CI) was computed to report the presence of association between the dependent and independent variables. Statistical significance was determined at a *P*-value of <0.05.

**Results:**

A total of 298 women participated in the study. In this study, the overall good knowledge of women regarding obstetrical and gynecologic surgical informed consent was 42.3% (95% CI: 36.7, 47.9). Urban residence (AOR = 2.32, 95% CI: 1.08, 5.03), educational status of women (AOR = 4.85, 95% CI: 1.99, 11.78), elective type of surgery AOR = 1.8, 95% CI: 1.14, 4.07), and having previous history of surgery (AOR = 7.2, 95% CI: 4.02, 15.15) were the identified determinant factors affecting knowledge of women toward obstetric and gynecologic surgical informed consent.

**Conclusion:**

In this study, more than half of the study participants lack overall good knowledge regarding obstetrical and gynecologic surgical informed consent. Residence, educational status, schedule of surgery, and previous history of surgery were the identified determinant factors affecting the knowledge of women toward obstetric and gynecologic surgical informed consent.

## Introduction

Informed consent (IC) is a principle in medical ethics and medical law that a patient must have adequate information and understanding before making decisions about their medical care. IC is a process of communication between the healthcare provider and the patient (1, 2). IC befalls when there is a covenant between the healthcare provider and patient on the benefits, possible complications, and alternative treatment options of the procedure (3).

The presence of a well-documented format tackles the ethical challenges of taking informed consent easily (4, 5). Brief IC information given to patients helps to know the risk–benefit analysis of their surgery (6). The IC should not be seen as simply formal legality but as an instrument to educate patients before undergoing surgery and increase their knowledge and involvement in the decision-making process (3). The knowledge of patients about IC saves health professionals from medicolegal issues (7). The IC is important for both clinicians and patients in surgery and should be seen as a usual activity to improve treatment outcomes (8).

The highest ethical principles of confidentiality ought to be pursued by all scientists, whose actions need to meet the criteria as set out by the International Society for Stem Cell Research, and informed consent has to be there clearly (9).

A study conducted in Italy showed that there was a double blind clinical trial on breast mammography examination for breast cancer, and it is very natural to have informed consent before the procedure (10). There is a study conducted in Italy on non-invasive prenatal test that clearly indicates how informed consent is very important for medical/surgical patient diagnosis and management (11).

A wide range of studies have tried to focus on possible different outcomes in terms of maternal–fetal health (12). Knowledge is one of the main intervention tools to improve patient comprehension in IC of obstetric and gynecologic surgical patients (13). Lack of knowledge about IC undermines patients' ethical clinical practice and queries the engrossment in the medical decision-making process (14). The low level (32.6%) of knowledge regarding IC for surgery hinders patients from choosing treatment options (15). The study emphasizes improving knowledge of obstetric and gynecologic surgical IC through planning and intervening strategies (16). A gap was identified in the delivery of patients' surgical IC in obstetrics and gynecologic preoperative procedures which in turn affects the quality of care (17). Healthcare providers should focus on maximizing the knowledge of patients about the IC process for obstetric and gynecologic surgical procedures (18).

The healthcare practitioners overestimate their patients' level of knowledge about the process of IC. This means that 30.7% of physicians said patients were informed about their clinical procedures in detail, but 11% of them were being informed deeply (19). Another study in Cairo concluded that there is a great discrepancy between health providers and patients about the knowledge of the IC process. Almost half (49.4%) of the healthcare providers said they inform patients about their medical conditions, while 38.9% of patients reported that they have been informed in detail (20). The information given to obstetric and gynecologic surgical patients before procedures was not adequate to fully aware of the choice they had made (21).

Only 14.5% of patients received their detailed procedure information before they underwent operation (22). Although the obstetric and gynecologic surgical patients had a good level of knowledge (97.5%) on IC, 31.7% of them had not signed the consent to undergo the procedure, and the main source of information was media (37.2%) and friends (37.1%). Here, 74.3% of the information about IC was not obtained from the signed clinicians (23). A study in Ethiopia scored a high level of satisfaction (98.1%) on patients’ preoperative surgical services and recommendations to emphasize the information given (24).

Approximately 10.5% of the respondents were knowledgeable about the surgical informed consents, and patients who had formal education and those living in urban areas were significantly associated with it (25). In assessing the knowledge of adult surgical patients’ IC, 36.09% had scored good knowledge, and for urban residents, having higher educational status and having a history of surgery were the factors affecting the knowledge of patients toward surgical IC (26). A systematic study done in Ethiopia on patients’ knowledge of surgical IC showed that 32% of respondents had appropriate knowledge (27). Another study in Jimma on knowledge of surgical IC and associated factors among patients who underwent obstetric and gynecologic surgery revealed that the overall knowledge of patients was 22.8. In this study, patient education, satisfaction level, and patient–provider relationship were the factors that affect knowledge of surgical IC (28).

In Ethiopia, little is known about the knowledge level and the determinant factors of patients toward obstetric and gynecologic surgical informed consent. In addition to this, as far as our knowledge is concerned, there was no study conducted in the study setting. Thus, this study aimed to assess the level of knowledge and associated factors of surgical informed consent among patients who underwent obstetric and gynecologic surgery at Debre Markos Comprehensive Specialized Hospital.

## Methods and materials

### Study design, area, and period

An institution-based cross-sectional study design was conducted at Debre Markos Comprehensive Specialized Hospital from 28 November to 30 December 2023. The hospital is located in Debre Markos town which is the capital city of East Gojjam Zone and is located 300 km northwest of Addis Ababa, the capital city of Ethiopia, and 265 km away from Bahir Dar, the capital city of the Amhara region. In the hospital, there are approximately 231 nurses, 51 midwives, 47 laboratory professionals, 45 pharmacists, 35 radiographers, 15 anesthetists, 3 integrated emergency surgical officers, 32 general practitioners, and 23 specialists. The hospital was assumed to serve five million people.

### Population

All obstetric and gynecologic surgical women who received services at Debre Markos Comprehensive Specialized Hospital during the data collection period were the study population. Obstetric and gynecologic surgical women aged above 18 years who received services at Debre Markos Comprehensive Specialized Hospital during the data collection period were included in the study.

### Sample size determination and sampling technique

The sample size was determined based on a single population proportion formula using the following assumptions: 5% margin of error, 95% confidence interval, and the proportion of knowledge from a previous study done at Jimma, which was 0.228 (28).

$$n=(z\alpha /2{)}^{2}p(1-p)/d^{2}=\frac{\left(1.96)2(0.228\times 0.772\right)}{(0.05)2}=271$$

After adding a 10% non-response rate, the final sample size became 298.

In this study, a systematic sampling technique was employed. The recent 1-month (423) operated patients were reported to get the interval size “*k*.” 423/298 = 1.42. Then every second operated patient was selected after randomly taking the first participants from 1 to 2.

### Measurements and operational definitions

The study consisted of six sociodemographic variables, five clinical-related variables, six service-related variables, and five perception questions. The outcome variable was knowledge (good/poor) of patients toward obstetric and gynecologic surgical informed consent. Thirteen knowledge assessment questions had “yes” or “no” responses. Patients who scored greater than or equal to the mean value of the knowledge assessment questions had good knowledge (28, 29). Similarly, patients who scored greater than or equal to the mean value of the perception assessment questions had good perception (18).

### Data collection tools and data quality control

A face-to-face interviewer-administered questionnaire was used to collect the data. The questionnaire was adapted from different literature previously studied and published as referenced here (18, 23, 28). Two data collector midwives were supervised by one MSc clinical midwife. The questionnaire was prepared first in English, then translated into the local language Amharic, and finally back-translated into English to check its consistency. One day of training was given to data collectors and supervisors. A pretest was done at Finote Selam General Hospital on 5% of obstetric and gynecologic surgical patients. During the data collection time, the questionnaire was checked in a daily manner, and corrections were made accordingly.

### Data processing and analysis

The collected data were coded, entered, and recorded. EpiData version 4.2 was used to enter the data and exported to SPSS version 25 for analysis. Binary logistic regression was used, and multivariable logistic regression was carried out for variables with a *P* < 0.25 in bivariable logistic regression to determine significant relationships between the dependent and independent variables. A *P*-value of <0.05 and a 95% confidence level were used to determine statistical significance. Reliability measures for knowledge and perception item scales were measured using Cronbach's alpha values with 0.83 and 0.81, respectively.

### Ethical consideration

This study was conducted in accordance with the Declaration of Helsinki. The ethical letter was obtained from the Institute Review Board of Debre Markos University Research Committee (protocol number: HSC/C/Scr/Co/6712) on 24 November 2023. Written informed consent was obtained before participants began the study. In addition to this, confidentiality was kept.

## Results

### Sociodemographic characteristics of the respondents

In this study, a total of 298 women whose surgery was done in the Debre Markos comprehensive specialized hospital were interviewed, making a response rate of 100%. Two hundred fifty-one (84.2%) respondents were in the age group of <34, with a mean age of 28.5 years. Approximately 225(76.5%) respondents lived in urban areas. Almost all (97%) respondents were orthodox Christian followers. Married women account for 280 (97.3%). When considering educational status, more than a third (37.6) of respondents attended college, and above. Among the total, 132 (44.3%) respondents were housewives (Table 1).

**Table 1:** Sociodemographic characteristics of women who underwent obstetric and gynecological surgery at Debre Markos Comprehensive Specialized Hospital, northwest Ethiopia, 2024 (*n* = 298).

Variables	Category	Frequency	Percentage
Age	<35 years	251	84.2
	35 and above years	47	15.8
Residence	Urban	225	75.5
	Rural	73	24.5
Religion	Orthodox	289	97
	Muslim	9	3
Marital status	Married	264	88.6
	Unmarried	34	11.4
Education	Cannot read and write	56	18.8
	Primary school	71	23.8
	Secondary school	59	19.8
	College and above	112	37.6
Occupation	Housewife	132	44.3
	Government employee	52	17.4
	Private employee	12	4
	Farmer	23	7.7
	Student	6	2
	Self-employee	15	5
	Daily laborer	6	2
	Health care provider	3	1
	Others	49	16.4

### Clinically related factors

This study also tried to identify the clinically related characteristics of the respondents. Accordingly, 251 (84.2%) respondents underwent emergency surgery. Twenty-four (8.1%) respondents had previous chronic medical diseases, of whom half (50%) were asthmatic and 37.5% were hypertensive. Near a third (29.5%) of respondents had at least one previous surgical history, of whom approximately 76.1%, 13.6%, and 10.2% of respondents had one, two, three, and above previous surgical histories, respectively (Table 2).

**Table 2:** Clinical-related factors of women who underwent gynecologic and obstetrical surgery at Debre Markos Comprehensive Specialized Hospital, northwest Ethiopia, 2024 (*n* = 298).

Variable	Category	Frequency	Percentage
Schedule of surgery	Elective	47	15.8
	Emergency	251	84.2
Previous chronic medical history	Yes	24	8.1
	No	274	91.9
Type of chronic medical disease	Hypertension	9	37.5
	Asthma	12	50
	Others	3	12.5
Previous surgical history	Yes	88	29.5
	No	210	70.5
Number of operations previously done	1	67	76.1
	2	12	13.6
	3 and above	9	10.2

### Patient-related factors

In this study, almost all (99%) respondents' informed consent for surgery was written in their mother tongue. The rest, three individuals (1%), took informed consent for surgery, but there were no written documents in the medical chart. Nearly two-thirds of respondents (62.8%) gave informed consent for surgery by midwives, and 106 (69.1%) respondents gave informed consent for surgery immediately before the surgery was done. Nearly half (47%) of respondents take <5 min to provide surgical informed consent. Regarding the time to make a decision, 91.6% of respondents decided early. Similarly, 287 (96.3%) respondents decided on their surgery by themselves (Table 3).

**Table 3:** Patient-related factors of women who underwent obstetric and gynecologic surgery at Debre Markos comprehensive specialized hospital, northwest Ethiopia, 2024 (*N* = 298).

Variables	Category	Frequency	Percentage
Consent written in the mother tongue	Yes	295	99
	No	3	1
The surgical informed consent process is explained by	Intern	84	29.2
	Midwife	187	62.8
	General practitioner doctor	24	8.1
Time of taking consent	The day before the date of surgery	28	9.39
	On the date of surgery	45	15.1
	Immediately before the surgery	206	69.1
	On the operating table	19	6.37
Time taken to provide informed consent	<5 min	140	47
	5–10 min	62	20
	>10 min	96	32.2
Time is taken into decision-making	Early	273	91.6
	Delay	25	8.4
Decision maker	Self	287	96.3
	Parent	8	2.7
	Spouse	3	1

### Perception of respondents toward surgical informed consent

In this study, one hundred seventy-eight (59.7%) respondents always trusted their surgeon during the time of surgery, while only eight respondents (2.7%) were never trusting their surgeon during the time of surgery. Similarly, one hundred seventy-five (58.1%) and eight (2.7%) respondents were always feeling comfortable with their surgeon and never feeling comfortable with their surgeon during the time of surgery, respectively. Nearly two-thirds (63.8%) of respondents felt respected by their surgeon; on the other hand, only five (1.7%) respondents felt never respected by their surgeon during the time of surgery. Similarly, one hundred ninety-three (64.7%) and eight (2.7%) respondents expressed their concern to their surgeon always and never, respectively. Lastly, one hundred eighty-eight (63.1%) respondents always felt that the surgeon heard, and understood, their opinion; on the other hand, only four (1.7%) respondents never felt that the surgeon heard and understood their opinion. Finally, the calculated Cronbach's alpha value of perception item scales was 0.81 (Table 4).

**Table 4:** Perception of informed consent among women who underwent obstetric and gynecologic surgery at Debre Markos comprehensive specialized hospital, northwest Ethiopia, 2024 (*n* = 298).

Variable	Never *N* (%)	Seldom *N* (%)	Sometimes *N* (%)	Often *N* (%)	Always *N* (%)
Trusting your surgeon	8 (2.7%)	9 (3%)	53 (17.8%)	50 (16.8%)	178 (59.7%)
Feeling comfortable with your surgeon	8 (2.7%)	5 (1.7%)	46 (15.4%)	66 (22.1%)	173 (58.1%)
Respecting your surgeon	5 (1.7%)	8 (2.7%)	28 (9.4%)	67 (22.5%)	190 (63.8%)
Expressing your concern to your surgeon	8 (2.7%)	2 (0.7%)	24 (8.1%)	71 (23.8%)	193 (64.7%)
The surgeon heard and understood your opinion	4 (1.7%)	8 (2.7%)	23 (7.7%)	75 (25.2%)	188 (63.1%)

### Overall perception

**Figure 1:**
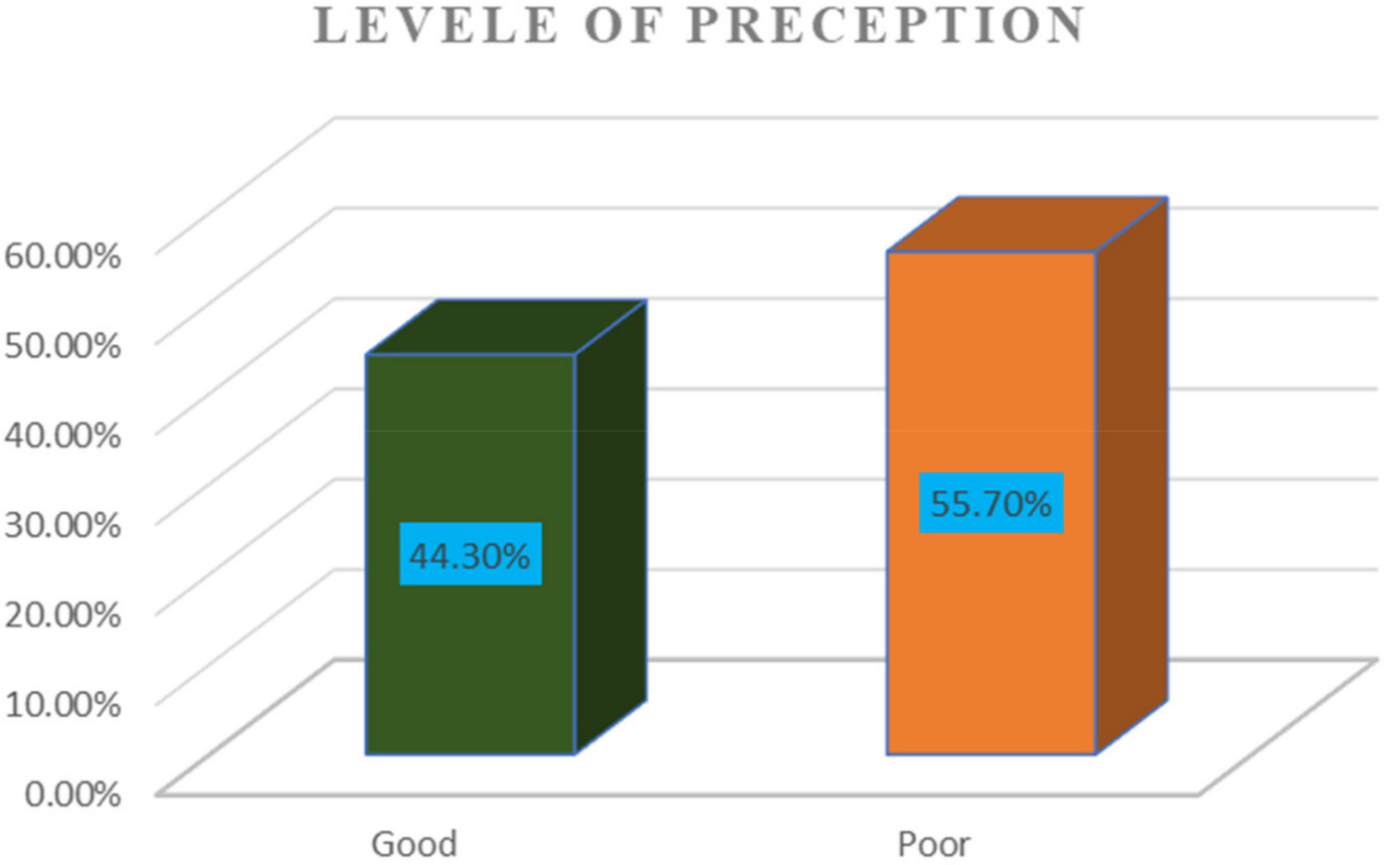
Overall perception of women toward informed consent among women who underwent obstetric and gynecologic surgery at Debre Markos Comprehensive Specialized Hospital, northwest Ethiopia, 2024 (298).

In this study, the overall perception level of women toward surgical informed consent was determined as good and poor perception. Accordingly, only 132 (44.3%) women had a good perception of surgical informed consent. Moreover, the overall mean value of perception of respondents toward surgical informed consent was 4.2 with an SD of 1.33 (Figure 1).

### Knowledge of respondents toward surgical informed consent

Reliability of knowledge measurement items was checked at Cronbach's alpha of 0.83. The mean values of overall knowledge were 4.22 with an SD of ±2. 78. In this study, only nine (3%) respondents read the written informed consent for their surgery. Nearly two-thirds (63.4%) of respondents did not know their surgeon during their surgery. Two hundred eighty (94%) respondents knew the reason for their surgery immediately before their surgery was done. Almost half (49%) of the respondent knew the type or nature of their surgery before their surgery was done. More than two-thirds (70.5%) and nearly half (45.3%) of respondents did not know the risks of anesthesia and complications of the surgery, respectively. Similarly, nearly two-thirds (61.4%) and more than two-thirds (74.8%) of respondents did not know the expected time taken for surgery and what to eat after surgery, respectively Table 5).

**Table 5:** Knowledge of women toward surgical informed consent among women who underwent obstetrical and gynecological surgery at Debre Markos Comprehensive Specialized Hospital, northwest Ethiopia, 2024 (*n* = 298).

Variables	Category	Frequency	Percentage
Signing a surgical informed consent	Yes	298	100
	No	0	0
Reading the written surgical informed consent	Yes	9	3
	No	289	97
Did you know the operative surgeon?	Yes	109	36.6
	No	189	63.4
Did you know the reason for the surgery?	Yes	280	94
	No	18	6
Did you know the type/nature of the surgery?	Yes	146	49
	No	152	51
Did you know about the options for alternative treatment?	Yes	62	20.8
	No	236	79.2
Did you know the risks of anesthesia during surgery?	Yes	88	29.5
	No	210	70.5
Did you know the risks and complications of surgery?	Yes	163	54.7
	No	135	45.3
Did you know the expected time of surgery?	Yes	115	28.6
	No	183	61.4
Did you know about postoperative care?	Yes	75	25.2
	No	223	74.8
Did you know what to eat after surgery?	Yes	126	42.3
	No	172	57.7
Do you know when to resume working?	Yes	79	26.5
	No	219	73.5
Did you know the cost of this treatment?	Yes	59	19.8
	No	239	80.2

### Overall knowledge of patients toward surgical informed consent

**Figure 2:**
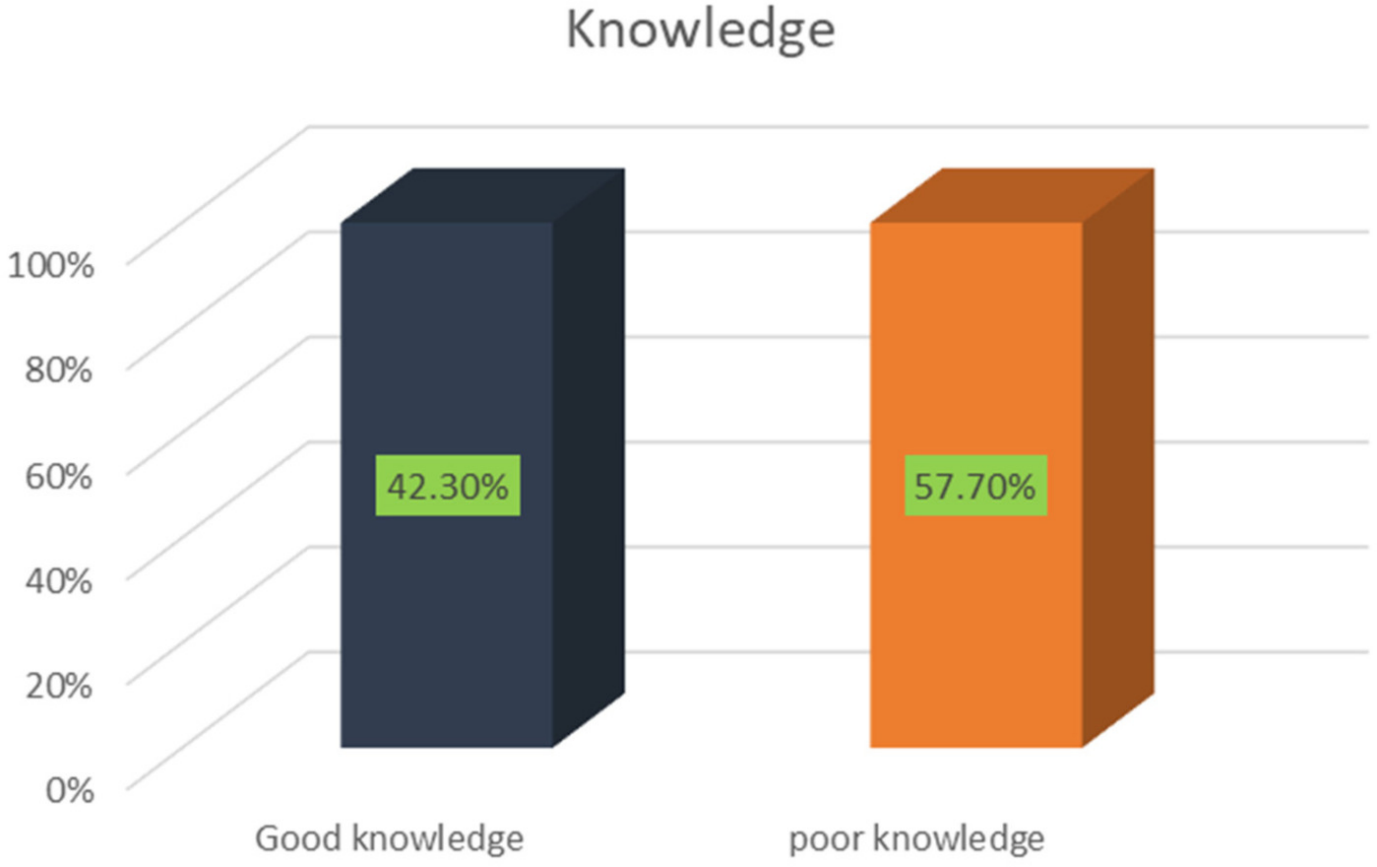
The overall knowledge status of women toward obstetrical and gynecological informed consent at Debre Markos Comprehensive Specialized Hospital in 2024.

In this study, the overall knowledge level of the study participants was determined as good vs. poor. Accordingly, the overall good knowledge of women toward surgical informed consent was 42.3% (95% CI: 36.7, 47.9). One hundred twenty-six (42.3%) respondents had good knowledge of surgical informed consent. On the other hand, more than half (57.7%) of study participants had poor knowledge of gynecologic and obstetrical surgery. The mean values of knowledge were also calculated. As a result, the overall mean value was 4.52 with an SD of 2.77 (Figure 2).

### Factors associated with knowledge of informed consent for gynecological and obstetrical surgery

In this study, the second aim was to identify the associated factors of the outcome variable. A variable with a *P*-value of ≤0.25 at 95% CI in bivariable binary logistic regression analysis was a candidate for multivariable logistic regression. Hence, the age of women, place of residence, educational status of women, schedule of surgery, previous history of surgery, time of taking informed consent, time of decision-making, time to decision-making, and perception of women toward surgical informed consent were variables fitted in the bivariable analysis at *P*-values of ≤0.25 to select the candidate variables for multivariable logistic regression. Finally, after adjusting the confounding variables, variables such as residence of women, educational status of women, schedule of surgery, and previous history of surgery were the identified predictors significantly associated with the outcome variable (Table 6). Accordingly, the odds of having good knowledge regarding informed consent were 2.3 times higher among women who live in urban areas as compared with their counterparts, adjusted odds ratio (AOR) = 2.32 (95% CI: 1.08, 5.03). Similarly, the odds of having good knowledge regarding informed consent were 4.8 times higher among women who attend college and above as compared with women who cannot read and write, AOR = 4.85 (95% CI: 1.99, 11.78).

**Table 6:** Factors associated with knowledge of women toward surgical informed consent among women who underwent obstetric and gynecologic surgery at Debre Markos Comprehensive Specialized Hospital in 2024.

Variables	Knowledge level		COR	AOR	*P*-value
	Good knowledge	Poor knowledge			
Age					
<35	122	129	2.47 (1.25, 4.90	1.79 (0.58, 5.51)	0.308
≥35	13	34	1	1	
Residence					
Urban	116	109	3.03 (1.69, 5.43)	**2.32 (1.08, 5.03)**	0.03*
Rural	19	54	1	1	
Education					
Cannot read and write	14	42	1	1	
Primary	20	51	1.18 (0.53, 2.6)	0.84 (0.32, 2.18)	0.718
Secondary	28	31	2.71 (1.23, 5.98)	2.39 (0.9, 6.29)	0.078
College and above	73	39	5.62 (2.74, 11.5)	**4.85** **(1.99, 11.78)**	≤0.001*
Schedule of surgery					
Elective	29	18	2.2 (1.16, 4.18)	**1.8** (**1.14, 4.07)**	0.025*
Emergency	106	145	1	1	
Previous surgical history					
Yes	71	17	9.53 (5.2, 17.4)	**7.2** (**4.02, 15.15)**	≤0.001*
No	64	146	1	1	
Time of taking informed consent					
The day before the date of surgery	18	10	2.17 (1.04, 8.6)	1.68 (0.84, 6.79)	0.076
On the day of surgery	20	25	0.89 (0.34, 3.6)	0.59 (0.12, 3.12)	0.543
Immediately before surgery	86	120	0.79 (0.47, 3.79)	0.69 (0.31, 3.62)	0.138
On the operating table	9	10	1	1	
Time of decision-making					
Early	122	151	0.75 (0.33, 1.69)	0.69 (0.22, 1.18)	0.526
Delay	13	12	1	1	
Time is taken into decision-making					
<5 min	47	51	0.85 (0.48, 1.5)	0.51 (0.24, 1.12)	0.093
5–10 min	38	66	0.53 (0.3, 0.93)	0.43 (0.33, 1.16)	0.423
>10 min	50	46	1	1	
Perception level					
Good	65	67	1.33 (0.84, 2.11)	1.26 (0.72, 2.02)	0.253
Poor	70	96	1	1	

Bold indicates statistically significant factors.

* *P* < 0.05.

In this study, the schedule of surgery was also another factor associated with the outcome variable. The odds of having good knowledge were 1.8 times higher among women whose surgery was done by elective as compared with women whose surgery was done by emergency, AOR = 1.8 (95% CI: 1.14, 4.07). Similarly, the odds of having good knowledge were seven times higher among women who had at least one previous surgical history as compared with women who had no history of previous surgery, AOR = 7.2 (95% CI: 4.02, 15.15).

## Discussion

This study aimed to assess the knowledge and associated factors toward surgical informed consent among women who underwent obstetrical and gynecologic surgery at Debre Markos Comprehensive Specialized Hospital. Accordingly, the overall knowledge of women toward surgical informed consent was 42.3% (95% CI: 36.7, 47.9). The finding of this study was in line with the study conducted at Arba Minch and Jinka Hospital (44%) (30). However, the finding of this study was lower than the study conducted in Turkey, 62% (7), and the study conducted in India, 54.17% (31). The discrepancy might be due to the difference in sociodemographic characteristics of study participants and variation in sample size. For instance, the socioeconomic status of Turkey is better than that of Ethiopia. In addition to this, sample size variation was also observed between the Ethiopian and Indian studies (398 and 298, respectively).

The finding of this study was also higher than those of the study conducted at Jimma Hospital, 22.8% (28); the study done in Rwanda, 17% (32); and the study conducted in Cairo, Egypt, 27.3% (20). The discrepancy might be due to the difference in sample size and sociodemographic variation between studies. For instance, the sample size in the study of Cairo, Egypt, was 216 which was lower than that in our study sample size which was 298.

The second objective of this study was to assess the associated factors affecting the knowledge of patients about obstetrical and gynecologic surgical informed consent. Accordingly, residence of women, educational status of women, schedule of surgery (elective vs. emergency), and previous history of surgery were the identified predictors significantly associated with the outcome variable.

The odds of having good knowledge of obstetric and gynecologic surgical informed consent were two times higher among women who live in urban areas compared with their counterparts. The finding of this study is supported by a similar study conducted in Nigeria (23). This might be because urban women were more exposed to media such as television and others, which increases women's knowledge of informed consent for surgery. The other explanation might be that urban women were more likely to be educated than rural women. Educational level affects the knowledge status of women who underwent surgery. In addition to this, urban women had high access to information regarding informed consent from different media sources.

Another determinant factor of women's knowledge of obstetrical and gynecological surgical informed consent was the educational status of women. Hence, the odds of having good knowledge of obstetric and gynecologic surgical informed consent were almost five times higher among women attending college and above as compared with women who cannot read and write. This study’s finding was similar to those of the studies conducted at Jimma (28) and Cairo, Egypt (20). The reason might be due to education increases women's awareness and deep understanding of something; for instance, in this case, it might increase the awareness and deep understanding of women toward their surgery and consent (26). The other explanations might be due to women being more educated (33) regarding the nature of the surgery, anesthesia complications, and the informed consent from different sources such as books, magazines, and newspapers.

The schedule of surgery (elective vs. emergency) was another determinant factor. Women whose surgery was performed by elective means were 1.8 times more likely to have good knowledge of informed consent as compared with their counterparts. This might be due to elective surgery; there may be enough time for the healthcare provider to give information regarding the informed consent and surgery for their patient (34). Getting more information to patients may increase their awareness and deep understanding regarding informed consent. This internship increases the level of knowledge of IC among patients who underwent obstetrical and gynecologic surgery. Similarly, having a previous history of surgery affects the level of patients’ knowledge of their informed consent. Accordingly, the odds of having good knowledge of obstetric and gynecologic surgical informed consent were almost seven times higher among women who had at least one previous surgical history as compared with women who had no previous surgical history. The possible reason for this might be due to patients who had a previous surgical history received postoperative healthcare education from their physician in the previous surgery. Especially, in the case of cesarean section, every woman might be getting health education during their antenatal care follow-up where and how they give childbirth (35). This might increase their knowledge regarding the nature of surgery to be done, and the possible complications of anesthesia and surgery; this might increase their overall knowledge regarding their surgical informed consent.

## Conclusion

This study revealed that patients’ overall knowledge of obstetrical and gynecological surgical informed consent was high relative to other literature conducted in Ethiopia. However, more than half lack adequate knowledge of obstetric and gynecologic surgical informed consent. The residence of women, educational status, schedule of surgery (elective vs. emergency), and previous history of surgery were the identified determinants of knowledge toward obstetrical and gynecologic surgical informed consent. Thus, healthcare providers shall focus on providing enough information regarding the nature of surgery, the complications of anesthesia, and the possible complications of the surgery for their patients. Providing enough information on the benefits and possible complications of surgery may help the patient to easily decide the choices of their surgical informed consent.

## Data Availability

The raw data supporting the conclusions of this article will be made available by the authors, without undue reservation.
